# The World’s Columbian Dental Meeting, 1893

**Published:** 1891-03

**Authors:** 


### The World's Columbian Dental Meeting, 1893.

The Executive Committee appointed at the last meeting of the Southern Dental Association and the American Dental Association to have full charge and make all arrangements for such a meeting, held its first regular session in Washington City, January 19th and 20th.

A careful consideration of the subject made it quite apparent that a great work is before the committee and those who may co-operate with them. In looking over the whole ground the committee became strongly impressed with the magnitude of the work before them, and with the necessity of securing the interest and co-operation of the entire profession.

About twenty general committees have been designated, and appointments made to some of them, especially to those whose work should begin at once, or as soon as possible. The names of these committees and the persons assigned upon them will be published in the next issue of the REGISTER.

In the committee there was the utmost unanimity and harmony of feelings on all matters pertaining to the great work to be accomplished. Every member seemed to realize that a great responsibility rests on this committee; one that no member can afford to neglect, nor withhold his best effort.

The next meeting will be held at Moorhead, North Carolina, August 11th, 1891.

